# Hearing, seeing, and feeling speech: the neurophysiological correlates of trimodal speech perception

**DOI:** 10.3389/fnhum.2023.1225976

**Published:** 2023-08-29

**Authors:** Doreen Hansmann, Donald Derrick, Catherine Theys

**Affiliations:** ^1^School of Psychology, Speech and Hearing, University of Canterbury, Christchurch, New Zealand; ^2^New Zealand Institute of Language, Brain and Behaviour, University of Canterbury, Christchurch, New Zealand

**Keywords:** audio-visual speech perception, audio-tactile speech perception, trimodal speech perception, multisensory integration, EEG, auditory evoked potentials

## Abstract

**Introduction:**

To perceive speech, our brains process information from different sensory modalities. Previous electroencephalography (EEG) research has established that audio-visual information provides an advantage compared to auditory-only information during early auditory processing. In addition, behavioral research showed that auditory speech perception is not only enhanced by visual information but also by tactile information, transmitted by puffs of air arriving at the skin and aligned with speech. The current EEG study aimed to investigate whether the behavioral benefits of bimodal audio-aerotactile and trimodal audio-visual-aerotactile speech presentation are reflected in cortical auditory event-related neurophysiological responses.

**Methods:**

To examine the influence of multimodal information on speech perception, 20 listeners conducted a two-alternative forced-choice syllable identification task at three different signal-to-noise levels.

**Results:**

Behavioral results showed increased syllable identification accuracy when auditory information was complemented with visual information, but did not show the same effect for the addition of tactile information. Similarly, EEG results showed an amplitude suppression for the auditory N1 and P2 event-related potentials for the audio-visual and audio-visual-aerotactile modalities compared to auditory and audio-aerotactile presentations of the syllable/pa/. No statistically significant difference was present between audio-aerotactile and auditory-only modalities.

**Discussion:**

Current findings are consistent with past EEG research showing a visually induced amplitude suppression during early auditory processing. In addition, the significant neurophysiological effect of audio-visual but not audio-aerotactile presentation is in line with the large benefit of visual information but comparatively much smaller effect of aerotactile information on auditory speech perception previously identified in behavioral research.

## 1. Introduction

Speech perception is a multimodal process. Listeners do not only rely on the auditory signal but also on the visual information provided by the speaker’s articulatory movements, especially when the acoustic signal is degraded. Sumby and Pollack (1954) laid the groundwork for this knowledge with their behavioral study. By presenting words in different noise environments, in auditory-only and audio-visual conditions, they demonstrated that as the acoustic signal became more degraded the audio-visual condition led to improved word intelligibility compared to the auditory-only condition. Further groundbreaking evidence of audio-visual interaction was provided by McGurk and MacDonald (1976) who showed that presenting mismatching stimuli (e.g., auditory /ba/ and visual /ga/) resulted in perception of a fused response /da/ (i.e., “McGurk effect”) (McGurk and MacDonald, 1976). The interference of visual information with auditory perception provided further evidence for the integration of bimodal information during speech perception. Since these seminal works, these findings have been extensively replicated and expanded on (for a review see Mallick et al., 2015).

In addition to the well-established influence of audio-visual presentation on speech perception, audio-tactile influences have also been demonstrated. Early behavioral evidence showed that feeling a speaker’s facial movements led to an enhancement of speech perception in deafblind perceivers (Alcorn, 1932) as well as trained healthy listeners (Reed et al., 1978, 1982). Further support for tactile influences on auditory speech perception comes from studies using vibro-tactile systems in deaf as well as normal-hearing adults (e.g., De Filippo, 1984; Weisenberger, 1989; Bernstein et al., 1991; Waldstein and Boothroyd, 1995). However, all these studies required extended participant training and used stimuli that are not representative of sensory information typically available during face-to-face interactions.

Evidence of audio-tactile integration in untrained subjects comes from behavioral studies investigating the effect of small air puffs (i.e., aerotactile information) on the listener’s skin (Gick and Derrick, 2009; Gick et al., 2010; Derrick and Gick, 2013). The air puff mimics a phonetic feature of specific speech sounds, distinguishing between high (e.g., /pa/) and low stop-release air flow (e.g., /ba/). By putting a hand in front of their face while saying /pa/, a native English speaker will experience a very noticeable burst of air. Doing the same with /ba/, they will experience low to unnoticeable air flow instead. Gick and Derrick (2009) applied air puffs on participants’ skin (either on their neck or hand) simultaneously with a degraded auditory signal. Their results showed that the presence of an air puff, unaffected by body location, enhanced correct identification of aspirated stimuli (e.g., /pa/) but it also interfered with correct identification of unaspirated stimuli (e.g., /ba/). These findings demonstrate that aerotactile information can interact with the auditory signal in a similar way as visual information in two-alternative forced choice experiments, however, this effect has not been observed in more complex tasks (Derrick et al., 2019a).

Interestingly, aerotactile information can also influence speech perception in the absence of auditory signals. In a study on visual-aerotactile speech perception, participants watched silent videos of a person saying /ba/ or /pa/, both alone or with air puffs applied to the skin (Bicevskis et al., 2016). They identified a syllable more likely as /pa/ when air puffs were present, demonstrating the integration of aerotactile information with visual information when an auditory signal is absent. This finding further confirms the multisensory nature of speech perception, with different bimodal sensory cues being integrated during the perception process.

Together, these studies demonstrated that speech perception can be a bimodal audio-visual and audio-tactile process, with some support for visuo-tactile speech perception. Derrick et al. (2019b) extended these findings to trimodal audio-visual-tactile integration in speech perception. In a two-way forced-choice auditory syllable-in-noise classification task (/pa/ or/ ga/), both visual and aerotactile information altered the signal-to-noise ratio (SNR) threshold for accurate identification of auditory signals. However, the strength of the effect of each modality differed. The visual component had a strong influence on auditory syllable-in-noise identification, resulting in a 28.0 dB improvement in SNR between matching and mismatching visual stimulus presentations. In comparison, the tactile component had a much smaller but significant influence, leading to a 1.6 dB SNR decrease in required auditory clarity. The results also showed that the three modalities provided additive influences. Visual and tactile information combined had a stronger influence on auditory speech perception than visual information alone, and the latter had a stronger influence than tactile stimuli alone. These findings demonstrate the simultaneous influence of both visual and tactile signals on auditory speech perception, illustrating a truly multimodal effect on behavioral responses.

To gain a better understanding of the processes contributing to multimodal speech perception, behavioral findings have been complemented by studies on neurophysiological processing. In an early EEG study, van Wassenhove et al. (2005) investigated the influence of audio-visual information on the N1 (negative peak approximately 100 ms following stimulus presentation) and P2 (positive peak approximately 200 ms following stimulus presentation) early cortical auditory event-related potentials (ERPs). In a three-alternative forced choice task, three syllables differing in visual saliency (/pa/, /ka/, and /ta/) were presented in matching and mismatching auditory, visual, and audio-visual conditions. Results showed that latencies of the auditory N1/P2 were reduced for audio-visual signals compared to the auditory-only condition, indicating faster auditory processing. The degree of visual saliency interacted with the temporal facilitation, with stronger visual predictors resulting in faster onset latencies (*p* < *t* < *k*). These findings were replicated in later studies (e.g., Arnal et al., 2009; Paris et al., 2016). van Wassenhove et al. (2005) also observed reduced N1/P2 amplitudes for audio-visual signals compared to auditory-only ones, independent of the saliency of the visual stimuli. They suggested that the N1/P2 suppression is independent of the featural content of speech input but rather reflects a more global bimodal integration process during which the preceding visual input leads to deactivation of the auditory cortices (van Wassenhove et al., 2005; see also Arnal et al., 2009; Baart et al., 2014).

A reduced and earlier N1/P2 complex in audio-visual compared to auditory-only conditions can also be observed for non-speech stimuli (e.g., handclapping; Stekelenburg and Vroomen, 2007; Vroomen and Stekelenburg, 2010). However, N1 suppression was only observed when visual information preceded and reliably predicted sound onset for both non-speech and speech stimuli, indicating that audio-visual N1 enhancement is dependent on anticipatory visual information. In addition, bimodal integration cannot only be observed for well-known or familiar perceptual experiences but also for audio-visual stimuli associated with less daily life experience (Paris et al., 2016; Treille et al., 2018). For example, Treille et al. (2018) used a facial view of lip movements or a sagittal view of tongue movements as visual stimuli. Both stimulus types interacted with the auditory speech signal, resulting in reduced P2 amplitude and latency in both audio-visual conditions compared to the auditory-alone one. This finding suggests that prior associative audio-visual experience is not necessary to result in bimodal interaction, and that dynamic and phonetic informational cues are sharable across modalities by relying on the listener’s implicit knowledge of speech production (Treille et al., 2018).

In contrast to studies on bimodal audio-visual processing, EEG studies focusing on audio-tactile effects are scarce. Treille et al. (2014a) compared the bimodal effects of audio-visual and audio-haptic speech perception. Participants, unexperienced with audio-haptic speech perception, were seated in front of the experimenter and had to keep their eyes closed while placing their right hand on the experimenter’s lips and cheek to feel the speech gestures. Using a two-alternative forced-choice task, two syllables (/pa/ or /ta/) were presented auditorily, visually and/or haptically. In line with previous research, the N1 amplitude was attenuated and its latency reduced during audio-visual compared to auditory-only speech perception. Tactile information also led to a speeding up of N1 in audio-haptic compared to auditory-only speech perception, indicating that tactile information can also accelerate this early auditory processing. As with visual information, articulatory movements and therefore tactile information precede the onset of the acoustic signal, which may lead to a speeding-up of N1 due to constraints put on subsequent auditory processing (van Wassenhove et al., 2005; Treille et al., 2014a). In a follow-up study, Treille et al. (2014b) also reported on haptically induced N1 amplitude suppression. However, this finding was stimulus dependent (i.e., /pa/ but not /ta/ and /ka/ syllables), possibly because the stronger saliency of the bilabial rounding movements for /pa/ conveyed a stronger predictive signal to facilitate the onset of the auditory event (Stekelenburg and Vroomen, 2007; Vroomen and Stekelenburg, 2010). Treille et al. (2014b) also reported shorter P2 latences in the audio-visual and audio-haptic compared to the auditory-only condition. This latency effect was independent of stimulus type or the degree of visual saliency, differing from earlier findings (van Wassenhove et al., 2005). The authors argued that differences between experimental settings and natural stimulus variability may be possible reasons for the different results.

Taken together, the neurophysiological findings showed that the auditory N1/P2 complex is modulated by information from different sensory modalities. While the evidence for audio-visual integration dominates, the limited audio-haptic findings indicate that tactile information is integrated in a similar manner as visual information, by contributing predictive information of the incoming auditory event. It is important to note that although the audio-haptic experience is less familiar or less natural than the audio-visual one, N1/P2 modulations could be observed suggesting that prior associative sensory experience is not needed for a noticeable audio-tactile interaction during early auditory processing. However, these findings have not yet been extended to more natural aerotactile stimuli. In addition, behavioral research showed a trimodal auditory-visual-tactile signal processing advantage compared to uni- and bi-modal speech stimuli (Derrick et al., 2019b), but we do not yet understand how the brain integrates all three modalities together. The current EEG study therefore aimed to identify whether (1) congruent audio-aerotactile speech signals led to neurophysiological processing advantages compared to auditory-only presentation, and (2) trimodal audio-visuo-aerotactile presentation of speech led to additional auditory processing enhancement beyond bimodally presented information. We hypothesized to see decreased amplitudes of the auditory N1 and P2 ERPs during matching audio-visual (AV) and audio-aerotactile (AT) stimulus presentation compared to auditory-only (A) stimuli, as well as an additional decrease in amplitude for trimodal signals (AVT) compared to bimodal speech stimuli. Based on behavioral findings (Derrick et al., 2019b), it was expected that the tactile effect would be smaller than the visual effect (i.e., AVT < AV < AT < A).

## 2. Materials and methods

### 2.1. Participants

Twenty adult New Zealand English speakers (3 males, 17 females, *M* = 23 years, *SD* = 4.8) were recruited. Participants completed a demographic information sheet and underwent an audiological screening. Pure tone audiometry testing was carried out for frequencies of 500 Hz, 1 kHz, 2 kHz, and 4 kHz using an Interacoustics AS608 screening audiometer. Average pure tone thresholds were calculated and if the threshold was less than or equal to 25 dB hearing level, hearing sensitivity was considered within normal range. None of the participants had a history of neurological disease or visual, speech, language, or hearing problems. Participants received a $20 voucher as compensation for their time. The study was approved by the University of Canterbury’s Human Ethics Committee (HEC 2017/23/LR-PS) and participants provided written informed consent.

### 2.2. Stimuli

#### 2.2.1. Recording of stimuli

**FIGURE 1:**
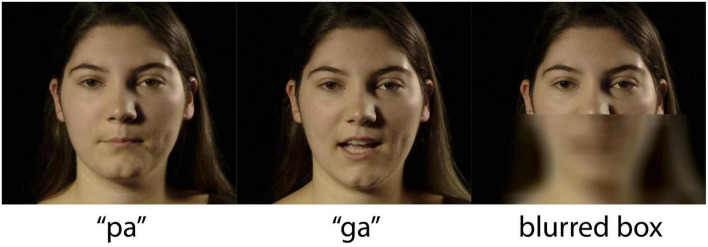
Screenshots of /pa/ and /ga/ video stimuli for the visual (V) conditions, and the blurred and still lower face for the auditory-only (A) condition. Reprinted with permission from Derrick et al. (2019b) Acoustic Society of America (ASA).

The stimuli in this experiment are a subset of the stimuli from Derrick et al. (2019a). One female speaker, producing forty tokens of /pa/ and /ga/ each, was recorded in a sound-attenuated room with a professional lighting setup. The video was recorded on a Sony MediaPro PMW-EX3 video camera set to record with the MPEG2 HD35 HL codec, with a resolution of 1920 by 1080 pixels (16:9 aspect ratio), a frame rate of 25 frames per second (fps), and a hardware-synched linear pulse-code-modulation (LPCM) 16-bit stereo audio recording at 48,000 Hz. The video was then converted to a time-preserving H.264 codec in yuv420p format encapsulated in an MP4 package, with audio extracted using FFMPEG (FFmpeg Developers, 2016). The audio was segmented in Praat (Boersma and Weenink, 2023), and the authors jointly selected ten recordings of each syllable that matched in duration, intensity, fundamental frequency, and phonation. In addition, the facial motion of each token was inspected to eliminate any case of eye-blink or noticeably distinguishable head motion. The video showed the complete face of the speaker, in frontal view from above the neck to the top of the head (see Figure 1).

#### 2.2.2. Creation of stimuli

The ten /pa/ and ten /ga/ tokens were sorted by length to form the closest duration-matched pairs. Software was written in R (R Development Core Team, 2018), WarbleR (Araya-Salas and Smith-Vidaurre, 2017), FFmpeg (FFmpeg Developers, 2016), and the Bourne Again Shell (BASH). The software took the timing of each video file and extracted the video with 750 ms lead time (prior the motion onset), and 500 ms follow time. For each video stimulus, it produced a version with right-channel audio from the original and left-channel audio that was either empty (for no air flow stimuli) or contained an 80 ms 12 kHz maximum intensity sine wave used to operate our custom air flow system. In addition to the audio-visual (AV) condition, for each video, a version was produced with a blurred and still lower face for the auditory-only (A) condition (see Figure 1). To generate speech noise, the recordings of the speech tokens were randomly superimposed 10,000 times within a 10 s looped sound file using an automated process written in R (R Development Core Team, 2018), WarbleR (Araya-Salas and Smith-Vidaurre, 2017), and FFMPEG (FFmpeg Developers, 2016). Noise created using this method results in a noise spectrum that is nearly identical to the long-term spectrum of the speech tokens from that speaker (Smits et al., 2004; Jansen et al., 2010). This type of noise has similar efficacy regardless of the volume at which it is presented, allowing for effective application of signal-to-noise ratios used in this experiment. The software then overlayed the right channel audio with speech-noise, making a video file for each token with SNRs of –8, –14, and –20 dB. The volume of the stimuli was kept constant at ∼60 dB, adjusting the level of white noise (Derrick et al., 2019b), ensuring that each token was of similar maximum amplitude for maximum comfort during the experiments.

#### 2.2.3. Presentation of stimuli

The experiment consisted of five conditions: auditory only, visual only, audio-visual, audio-tactile, and audiovisual-tactile. Bimodal and multimodal conditions were only presented in a congruent context (e.g., air flow only for /pa/ tokens but not for /ga/ tokens) leading to eight different types of stimuli across the five conditions. The visual-only condition was presented with a constant white noise at ∼60 dB, other conditions were presented at three different SNRs (–8, –14, and –20 dB). For each item 50 trials were presented, leading to a total of 1,000 stimulus presentations over the entire EEG recording session (see Table 1).

**TABLE 1:** Type and number of trials per modality and SNR (total of 1,000 trials presented over 10 blocks).

Modalities/SNRs	−8 dB	−14 dB	−20 dB
**Audio only (A)**			
pa	50	50	50
ga	50	50	50
**Audio-visual (AV)**			
pa	50	50	50
ga	50	50	50
**Audio-tactile (AT)**			
pa	50	50	50
**Audio-visual-tactile (AVT)**			
pa	50	50	50
**Visual only (V)**	**Noise**		
pa	50		
ga	50		

**FIGURE 2:**
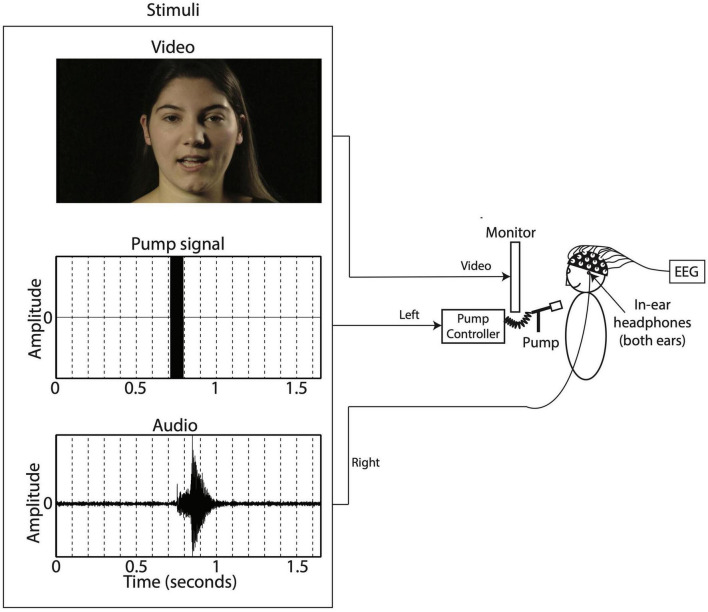
Experimental setup.

During the auditory-only condition the speaker was presented with a blurred rectangle covering the lower face and articulatory movements (see Figure 1). For the bi- and trimodal conditions, the auditory, tactile and visual signals were presented simultaneously (Derrick et al., 2009). Figure 2 shows a schematic overview of the experimental setup. Sound was presented through EARtone 3A Insert Headphones in both ears at ∼60 dB, simultaneous with the relevant video. Visual stimuli were displayed on a computer screen placed 1 m in front of the participant. The tactile signal involved a slight, inaudible, cutaneous air flow presented on the suprasternal notch of the participant via the Murata piezoelectric air pump that was positioned 3 cm in front of the individual. The 80 ms 12 kHz sine wave was used to operate our air flow production system (Derrick and De Rybel, 2015). The air flow system uses a Murata’s microblower, a 20 mm × 20 mm × 1.85 mm piezoelectric air pump with up to 0.8 l/m flow, max 19.38 cm/H_2_O pressure, and approximately 30 ms 5–95% intensity rise time, allowing artificial approximation of continuously varying air flow in speech (Derrick and De Rybel, 2015). The sine wave in the left channel turns on the air flow system at full capacity, generating its highest air flow with a duration within the range of the voice onset time of a word-onset velar voiceless stop (/ga/), and at the long end of length for that of a labial voiceless stop (/pa/) (Lisker and Abramson, 1967).

### 2.3. EEG recording and procedure

Participants were given a two-alternative forced-choice task (2AFC). They were told that they may perceive some noise and puffs of air along with the speech syllables they needed to identify. EEG data were continuously recorded using a 64-channel BioSemi Active Two system (Brain Products GmbH, 2021, München, Germany). Data were sampled at 250 Hz. Electrooculograms (EOG) were recorded from individual electrodes placed next to the left and right eye for horizontal eye movements and above and below the left eye for vertical eye movements. Two additional electrodes were placed on the left and right ear lobes for off-line referencing. During the EEG recording, participants were seated in a soundproof booth. The presentation of trials was coordinated with the E-prime 2.0 software (E-Studio; version 2.0, Psychology Software Tools, Inc., Pittsburgh, PA, USA). Each trial began with the presentation of a black fixation cross in the middle of a light gray background, which was shown for 120 ms. Then a stimulus from one of the five conditions (A, V, AV, AT, and AVT) was presented playing for 2,000 ms. A 320 ms pause followed in order to avoid possible interference between speech identification and motor response induced by the participants’ button press (Ganesh et al., 2014). Then a question mark symbol appeared on the screen and participants were required to categorize each syllable by pressing on one button corresponding to /pa/ or /ga/ (counterbalanced between subjects) on the response box with no forced time limit. After participants selected an answer, they were given a blank screen during the inter-trial interval, randomized between 800 and 1000 ms.

Participants were given three practice items prior to starting the actual experiment consisting of 10 blocks of 100 trials each. Trials within blocks were randomized across modalities but stimuli from the same modality were not presented for more than two times consecutively. The order of blocks was randomized for each participant. After each block participants took a two- to 3-min rest and continued the experiment by pressing the spacebar. The experiment lasted approximately 1.5 h, including subject preparation, explanations and pauses between blocks.

### 2.4. Data analyses

Three participants were excluded from data analysis due to technical problems during the EEG recording. For the remaining 17 participants offline data analysis was conducted using BrainVision Analyzer 2.0 (Brain Products GmbH, 2021, München, Germany). Data was re-referenced to the averaged voltage of the two earlobe electrodes and bandpass-filtered to 0.1 to 30 Hz (slope: 24 dB/octave). The signal was then segmented into 1,100-ms-long epochs starting 100 ms pre-stimulus until 1,000 ms post-stimulus. Only trials with correct responses were included in further EEG analyses. A semi-automated routine with additional visual inspection was used to exclude epochs that contained artifacts (voltages exceeding ± 100 μV, at any channel). The mean artifact rate was 11%. Epochs were baseline corrected using the EEG data from −100 to 0 ms relative to stimulus onset. Averaged ERPs for the conditions A, AV, AT, and AVT were calculated for each participant. The Cz electrode was used for ERP analysis as in previous reports (Baart et al., 2014, 2017; Baart, 2016). Based on visual inspection of the grand average waveforms, a time window from 100 to 250 ms was selected that encompassed the auditory N1 and P2 components. The averaged EEG activity was extracted from three 50 ms time bins (e.g., Schepers et al., 2013; Baart and Samuel, 2015). The multimodal integration effects on N1 and P2 were analyzed by comparing A, AV, AT, and AVT responses (Baart, 2016). Statistical analyses were carried out using a three-way repeated measures ANOVA for each syllable type (/ga/ and /pa/) with time window, modality and SNR as within-subject factors. An alpha level of 0.05 was used to determine statistical significance. Bonferroni’s correction was applied in further *post hoc* analyses.

Behavioral data was recorded during the EEG experiment in the form of accuracy data. A two-way repeated measures ANOVA for each syllable type (/pa/ and /ga/) was conducted with modality and SNR as within-subject factors. Greenhouse–Geisser correction was used whenever sphericity was violated and Bonferroni’s correction in further *post hoc* analyses. Significance was defined at the *p* < 0.05 level.

## 3. Results

### 3.1. Behavioral data

#### 3.1.1. Accuracy /pa/ syllable

**FIGURE 3:**
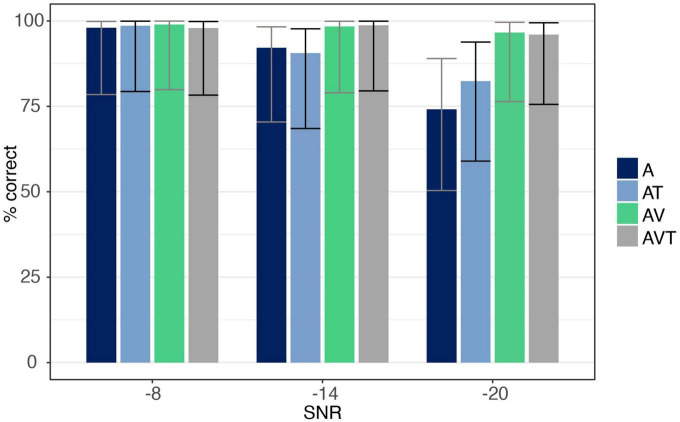
Accuracy data for syllable /pa/ for auditory-only (A), audio-visual (AV), audio-tactile (AT), and audio-visual-tactile (AVT) conditions at each SNR level (–8, –14, –20 dB). Error bars are based on Binomial confidence intervals (95%).

The number of correct trials under each condition are reported in Figure 3. ANOVA showed a main effect for both *SNR* [*F*_(1.18,18.94)_ = 37.33, *p* < 0.001, η*_*p*_*^2^ = 0.70], and *modality* [*F*_(1.89,30.28)_ = 17.29, *p* < 0.001, η*_*p*_*^2^ = 0.52], with AV and AVT conditions being identified more accurately than the A only (all *post hoc* analyses *p* < 0.001), and the AT conditions (all *post hoc* analyses *p* < 0.001). The interaction between *SNR and modality* was also significant [*F*_(2.29,36.65)_ = 15.05, *p* < 0.001, η*_*p*_*^2^ = 0.49], revealing modality effects at the −14 and −20 dB SNR level but not at −8 dB. At −14 dB SNR, listeners responded to AV and AVT trials more correctly than to A (*post hoc* analyses *p* = 0.015 and *p* < 0.01, respectively) and AT trials (*post hoc* analyses *p* = 0.003 and *p* < 0.001, respectively). The same was observed at the −20 dB level, with AV and AVT showing more accuracy than A only (all *post hoc* analyses *p* < 0.001) and AT (all *post hoc* analyses *p* = 0.002). No difference in accuracy was found between A and AT at −14 and −20 dB (*post hoc* analyses *p* = 1.0 and *p* = 0.61, respectively).

#### 3.1.2. Accuracy /ga/ syllable

**FIGURE 4:**
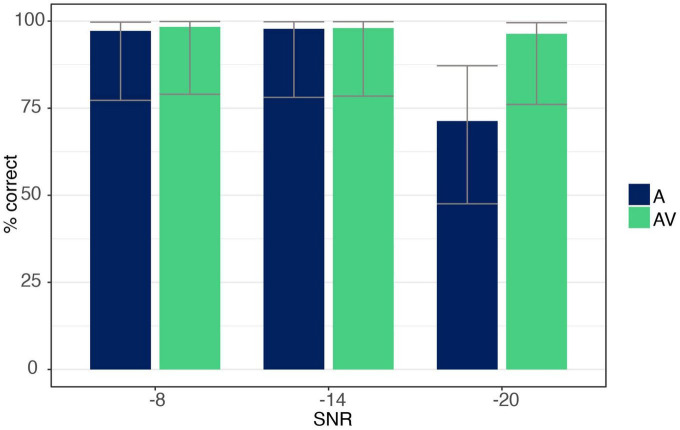
Accuracy data for syllable /ga/ for auditory-only (A) and audio-visual (AV) conditions at each SNR level (–8, –14, –20 dB). Error bars are based on Binomial confidence intervals (95%).

The ANOVA showed a main effect for both *SNR* [*F*_(1.09,17.53)_ = 39.91, *p* < 0.001, η*_*p*_*^2^ = 0.71] and *modality* [*F*_(1,16)_ = 50.74, *p* < 0.001, η*_*p*_*^2^ = 0.76], with listeners identifying trials more accurately in the AV compared to the A condition. The ANOVA further revealed an interaction effect between *SNR and modality* [*F*_(1.17,18.79)_ = 44.63, *p* < 0.001, η*_*p*_*^2^ = 0.74], indicating a difference between modalities only at the −20 dB level with listeners responding more correctly when additional visual information was present (*post hoc* analysis *p* < 0.001; see Figure 4).

### 3.2. ERP data

**FIGURE 5:**
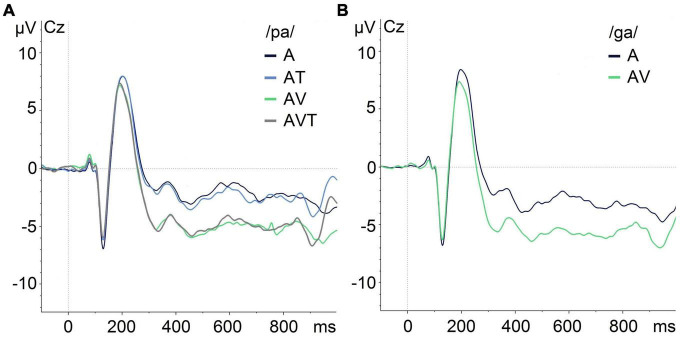
Grand-average of auditory evoked potentials for /pa/ and /ga/ syllables at Cz in different modalities illustrating the N1/P2 effects. **(A)** ERPs for /pa/ in auditory-only (A), audio-tactile (AT), audio-visual (AV), and audio-visual-tactile (AVT) conditions. **(B)** ERPs for /ga/ during auditory-only (A) and audio-visual (AV) conditions.

Figure 5 shows the grand averaged responses obtained for each modality. Visual inspection indicated a reduced amplitude of the audio-visual and audio-visual-tactile N1 and P2 auditory ERPs compared to the auditory-only condition, which was confirmed by the statistical analyses. Visual inspection also indicated a reduced N1 amplitude in audio-tactile compared to audio-only condition for /pa/. However, this difference was not statistically significant.

#### 3.2.1. 100–250 ms time window: /pa/ syllable

The ANOVA showed a significant main effect for *time window* [*F*_(2,32)_ = 68.13, *p* < 0.001, η*_*p*_*^2^ = 0.81], and an interaction with *modality* [*F*_(6,96)_ = 13.86, *p* < 0.001, η*_*p*_*^2^ = 0.46]. No other statistically significant main effects or interactions were present. *Post hoc* comparisons testing the four modalities (A, AV, AT, AVT) against each other within each time window, showed a main effect of *modality* in the 100–150 ms time window, *F*_(3,150)_ = 5.25, *p* = 0.002, η*_*p*_*^2^ = 0.09, and the 200–250 ms time window, *F*_(3,150)_ = 12.79, *p* < 0.001, η*_*p*_*^2^ = 0.20. In the 100–150 ms time window, A (*M* = −3.99 μV, *SD* = 2.95) showed a more negative (i.e., larger) amplitude than AV (*M* = −3.15 μV, *SD* = 3.14) and AVT (*M* = −3.14 μV, *SD* = 3.21). No significant difference was found for A (*M* = −3.99 μV, *SD* = 2.95) compared to AT (*M* = −3.49 μV, *SD* = 3.01; *p* = 0.17). In the 200–250 ms time window, A (*M* = 6.30 μV, *SD* = 3.10) showed a more positive (i.e., larger) amplitude than AV (*M* = 4.78 μV, *SD* = 2.93) and AVT (*M* = 4.69 μV, *SD* = 3.17). In addition, AT (*M* = 6.19 μV, *SD* = 2.86) resulted in a more positive amplitude than AV (*M* = 4.78 μV, *SD* = 2.93) and AVT (*M* = 4.69 μV, *SD* = 3.17). No significant difference was found for A (*M* = 6.30 μV, *SD* = 3.10) compared to AT (*M* = 6.19 μV, *SD* = 2.86) in this time window (*p* = 1.0). Similarly, no significant differences were identified in the 150–200 ms time window [*F*_(3,150)_ = 0.75, *p* = 0.52, η*_*p*_*^2^ = 0.01].

#### 3.2.2. 100–250 ms time window: /ga/ syllable

The ANOVA revealed a significant main effect for *time window* [*F*_(2,32)_ = 60.46, *p* < 0.001, η*_*p*_*^2^ = 0.79], and an interaction with *modality* [*F*_(2, 32)_ = 11.22, *p* < 0.001, η*_*p*_*^2^ = 0.41]. No other significant main effects or interactions were found. *Post hoc* pairwise comparisons showed only a significantly reduced amplitude for AV (*M* = 4.89 μV, *SD* = 3.02) compared to A (*M* = 6.57 μV, *SD* = 3.18) in the 200–250 ms time window, *t*(50) = 4.34, *p* < 0.001.

## 4. Discussion

Previous EEG research showed that speech perception is a multimodal process, demonstrating a neurophysiological processing advantage for bimodal audio-visual and audio-haptic signals over auditory-only ones (van Wassenhove et al., 2005; Pilling, 2009; Treille et al., 2014a,b). The present study aimed to expand previous work by investigating bimodal audio-aerotactile as well as trimodal audio-visuo-aerotactile integration in speech perception. Given that this study is the first to investigate the influence of aerotactile and trimodal stimuli, we used a basic EEG paradigm with a two-alternative forced-choice identification task, presenting two syllables (/pa/ and /ga/) at three different noise levels.

The main findings of our study showed that presenting congruent visual information led to significant amplitude reductions compared to auditory-only information, whereas the presentation of aerotactile speech signals did not lead to similar neurophysiological processing advantages in the auditory N1/P2 complex. Comparison of the trimodal AVT with the AV condition further confirmed the negative result related to the presentation of air puffs. Our results therefore did not confirm our hypothesis of a decrease in amplitudes with the addition of modalities (i.e., AVT < AV < AT < A). This is consistent with the behavioral /pa/ findings at −20 dB SNR in the current study, showing no significant difference in the audio-tactile compared to auditory-only condition, while the increase in accuracy in the two conditions with additional visual information was statistically significant. These findings are in line with previous behavioral trimodal speech perception results that showed a very small—albeit statistically significant–effect of aerotactile information compared to a strong influence of visual information on auditory syllable-in-noise identification (Derrick et al., 2019b).

As auditory, visual and aerotactile speech outputs share information in optimal environments, benefits of multimodal presentation become evident when one of the signals is degraded (e.g., Sumby and Pollack, 1954; Gick and Derrick, 2009). The relatively small behavioral effect of aerotactile information in previous studies (Derrick et al., 2019a,b) and absence of significant behavioral and neurophysiological effects of this type of information in the current study, suggests that aerotactile stimuli may only significantly enhance speech perception when there is no redundancy between this signal and all other information available.

The insignificant effect of the aerotactile information can further be interpreted in the context of existing EEG research investigating hand-to-face speech perception. Treille et al. (2014b) compared AV and audio-haptic (AH) modalities and reported a reduced N1 amplitude in both AV and AH modalities compared to the A modality. Importantly, the attenuation of N1 in the AH modality was restricted to the syllable /pa/ (not /ta/ or /ka/) due to the dependency of the N1 amplitude on the temporal relationship of sensory input. Due to the haptic saliency of the bilabial rounding movements involved in /pa/, this syllable was more reliable in predicting the sound onset than /ta/ and /ka/(Treille et al., 2014b). In the present study the air puff and auditory event were perceived simultaneously (Derrick et al., 2009), thus no anticipatory cue was available in the tactile signal unlike in the haptically perceived /pa/. This could explain the absence of an N1 amplitude attenuation for the aerotactile signal and would be in line with past AV studies showing that N1 suppression depends on the leading visual signal and how well it predicts the onset of the auditory signal (Stekelenburg and Vroomen, 2007; Vroomen and Stekelenburg, 2010; Baart et al., 2014). It is worth noting that in the grand-average data (Figure 5) the tactile component does appear to have a visible effect on the N1 component of the auditory evoked potential. Although the visual effect is stronger, tactile induced suppression could indicate a potential processing advantage of AT over A, which would imply integration of tactile information regardless of its effectiveness in predicting sound onset (van Wassenhove et al., 2005). However, this remains highly speculative at this stage and requires further investigation.

Our AV compared to A results are consistent with previous studies reporting decreased auditory-evoked N1 and P2 ERPs when visual information accompanied the acoustic signal (Klucharev et al., 2003; Besle et al., 2004; van Wassenhove et al., 2005; Brunellière et al., 2013), confirming that visual information evoked an amplitude suppression during early auditory processing. For /pa/, AV signals yielded less negative ERPs in the 100–150 ms window (i.e., N1) and less positive ERPs in the 200–250 ms window (i.e., P2) compared to A signals. For /ga/ the AV compared to A amplitude reduction was only observed in the 200–250 ms window. A similar finding has been reported by Baart et al. (2014). Using stimuli that started with an alveolar instead of labial place of articulation, they observed an attenuation of the auditory P2 amplitude but not of the N1 amplitude. As the N1 amplitude is sensitive to a temporal relationship between visual and auditory signals (Stekelenburg and Vroomen, 2007; Vroomen and Stekelenburg, 2010), N1 suppression is dependent on anticipatory visual motion and its prediction of sound onset. Based on these findings, Baart et al. (2014) suggested that the temporal prediction of sound onset in alveolar stimuli may be less effective compared to stimuli that start with a labial place of articulation, resulting in a lack of N1 amplitude reduction. As /ga/ has a velar place of articulation, the absence of an attenuated N1 could be attributed to a less effective visual signal in the prediction of sound onset compared to the labial /pa/. Of note, however, is that other studies have reported N1 amplitude reductions for both labial and velar stimuli (van Wassenhove et al., 2005; Brunellière et al., 2013; Treille et al., 2014b). Inconsistencies of N1 and P2 effects across studies have been attributed to different factors, including variability in experimental tasks and associated cognitive load, experimental settings, data processing and analyses [see Baart (2016) for review and meta-analysis].

Our behavioral results showed a significant interaction effect between modality and SNR level, similar to previous behavioral research showing increased reliance on the visual signal as speech becomes more degraded (e.g., Sumby and Pollack, 1954; Derrick et al., 2019b). In contrast, the EEG results did not show the same interaction effect. The lack of a significant effect of varying noise levels in the current study could be attributed to the use of a simple two-alternative forced-choice speech identification task. Visual information seemed to dominate prediction of the incoming syllable no matter how much noise obscured the auditory signal. Similar findings have been reported previously. For example, Gilbert et al. (2012) also used a two-alternative forced-choice task (/ba/ vs /ga/) in four different acoustic environments (quiet, 0 dB SNR, –9 dB SNR, –18 dB SNR) and did not find an effect of SNR on audio-visual speech perception. Use of a larger sample of syllables would avoid using an elimination strategy and should be considered in future experiments (Liu et al., 2013).

While the airflow pump used in the current study has successfully been used in previous bi- and tri-modal behavioral aerotactile research (Derrick et al., 2019a,b), a limitation of this artificial air flow pump is that produces the same pressure (max 1.5 kPa) as speech but only one twelfth of the air flow (0.8 l/m) that speech normally generates (11.1 l/m) (Derrick et al., 2019a). To address the lower air flow, the system was placed close to the skin (3 cm away from participant) capable of covering a smaller area of skin compared to what a speaker’s breath covers from a close speaking distance. This smaller impact area may negatively affect skin mechanoreceptor response among the Fast-acting type II receptors (Pacinian corpuscles), which only require a tiny 0.01 mm of skin indentation to respond, but must be impacted over an area about the size of a hand (Johnson, 2002). In addition, recent work on speech air flow shows that air flow patterns in speech are produced with much more widely varying penetration speeds than previously recognized (see Derrick et al., 2022). Future studies with new state of the art air flow systems may be able to address these factors.

In order to achieve our goals without placing an excessive burden on participants (>1.5 h of EEG recording), we kept the paradigm as simple as we could. This entailed using a limited number of trails to represent each stimulus (50 trials per modality and per SNR, respectively), which is a limitation. Future studies considering a larger number of trials or a more complex paradigm could provide more insights into the relevance of aerotactile information during early auditory processing in speech perception. Future studies may also consider the analysis of visual-only and tactile-only modalities to test predictions in an additive model framework (e.g., AT-T ≠ A).

## 5. Conclusion

We reported the first EEG study to investigate the effect of bimodal audio-aerotactile and trimodal audio-visuo-aerotactile information on early auditory processing. Our findings provided support for the facilitation of auditory processing following presentation of congruent visual information. Our results did not confirm our hypothesis of an additional beneficial effect of aerotactile information on the neurophysiological processing of auditory signals. Together, the present findings confirm the large benefit of visual information on auditory speech perception in noise and are in line with the comparatively much smaller effect of aerotactile information identified in previous behavioral research.

## Data availability statement

The original contributions presented in the study are included in the article, further inquiries can be directed to the corresponding author.

## Ethics statement

The studies involving humans were approved by Human Ethics Committee, University of Canterbury, Christchurch, New Zealand. The study was conducted in accordance with the local legislation and institutional requirements. The participants provided their written informed consent to participate in this study. Written informed consent was obtained from the individual(s) for the publication of any potentially identifiable images or data included in this article.

## Author contributions

DH mainly responsible for data collection, data analysis, and manuscript writing. DD and CT wrote sections of the manuscript. DH and DD mainly responsible for production of figures and table. All authors contributed to conception and design of the study, manuscript revision, read, and approved the submitted version.
